# Seeking the state of the art in standardized measurement of health care resource use and costs in juvenile idiopathic arthritis: a scoping review

**DOI:** 10.1186/s12969-019-0321-x

**Published:** 2019-05-06

**Authors:** Michelle M. A. Kip, Gillian Currie, Deborah A. Marshall, Luiza Grazziotin Lago, Marinka Twilt, Sebastiaan J. Vastert, Joost F. Swart, Nico Wulffraat, Rae S. M. Yeung, Susanne M. Benseler, Maarten J. IJzerman, on behalf of the UCAN CAN-DU Health Economics Working Group, Gouke Bonsel, Brian M. Feldman, Esther Hoppenreijs, Bianca Lang, Claire LeBlanc, Ana Sepulveda, Karine Toupin-April, Philomine van Pelt, Annet van Royen-Kerkhof

**Affiliations:** 1 Department of Health Technology and Services Research, Faculty of Behavioural, Management and Social Sciences, Technical Medical Centre, University of Twente, P.O. Box 217, 7500 AE Enschede, the Netherlands; 2 Department of Community Health Sciences, Cumming School of Medicine, University of Calgary, Calgary, Alberta Canada; 3 Department of Paediatrics, Cumming School of Medicine, University of Calgary, Calgary, Alberta Canada; 4 Division of Rheumatology, Department of Pediatrics, Alberta Children’s Hospital, Cumming School of Medicine, University of Calgary, Calgary, Alberta Canada; 5 Division of Paediatrics, Department of Paediatric Rheumatology, University Medical Center Utrecht, Wilhelmina Children’s Hospital, Utrecht, The Netherlands; 6 Division of Rheumatology, Department of Paediatrics, The Hospital for Sick Children, University of Toronto, Toronto, Ontario Canada

**Keywords:** Health resources, Health care costs, Juvenile idiopathic arthritis, Review

## Abstract

**Background:**

This study aims to describe current practice in identifying and measuring health care resource use and unit costs in economic evaluations or costing studies of juvenile idiopathic arthritis (JIA).

**Methods:**

A scoping review was conducted (in July 2018) in PubMed and Embase to identify economic evaluations, costing studies, or resource utilization studies focusing on patients with JIA. Only English language peer-reviewed articles reporting primary research were included. Data from all included full-text articles were extracted and analysed to identify the reported health care resource use items. In addition, the data sources used to obtain these resource use and unit costs were identified for all included articles.

**Results:**

Of 1176 unique citations identified by the search, 20 full-text articles were included. These involved 4 full economic evaluations, 5 cost-outcome descriptions, 8 cost descriptions, and 3 articles reporting only resource use. The most commonly reported health care resource use items involved medication (80%), outpatient and inpatient hospital visits (80%), laboratory tests (70%), medical professional visits (70%) and other medical visits (65%). Productivity losses of caregivers were much more often incorporated than (future) productivity losses of patients (i.e. 55% vs. 15%). Family borne costs were not commonly captured (ranging from 15% for school costs to 50% for transportation costs). Resource use was mostly obtained from family self-reported questionnaires. Estimates of unit costs were mostly based on reimbursement claims, administrative data, or literature.

**Conclusions:**

Despite some consistency in commonly included health care resource use items, variability remains in including productivity losses, missed school days and family borne costs. As these items likely substantially influence the full cost impact of JIA, the heterogeneity found between the items reported in the included studies limits the comparability of the results. Therefore, standardization of resource use items and unit costs to be collected is required. This standardization will provide guidance to future research and thereby improve the quality and comparability of economic evaluations or costing studies in JIA and potentially other childhood diseases. This would allow better understanding of the burden of JIA, and to estimate how it varies across health care systems.

**Electronic supplementary material:**

The online version of this article (10.1186/s12969-019-0321-x) contains supplementary material, which is available to authorized users.

## Background

Juvenile idiopathic arthritis (JIA) is the most common chronic rheumatologic disease in childhood [1]. Children with JIA suffer from joint inflammation, stiffness, contractures, and pain, which can lead to fatigue, growth abnormalities and functional impairment [2–5]. Due to its chronic nature, children diagnosed with JIA are at higher risk of developing behaviour and/or psychiatric disorders (e.g. social isolation, depression, anxiety) [6, 7], they miss more school or work days due to symptoms or medical appointments [8, 9], and have lower Health-Related Quality of Life (HRQoL) [6] than their peers. In addition, there is an associated risk of developing extra-articular diseases such as uveitis, which may lead to blindness if left undiagnosed or untreated [2]. Treatment of JIA consists of a combination of pharmacological, physical and occupational therapy, and psychological support [3]. In patients with active disease or with inadequate response to conventional pharmacological treatment (e.g. MTX, NSAIDs and DMARDs), the use of a (much more expensive) biologic DMARD is recommended [4, 10, 11].

Besides the direct negative health impact of JIA, it may result in lifelong functional limitations [12], lower educational attainment [13], higher unemployment rates [13] and lower HRQoL [14, 15]. In addition, JIA does not only affect the patient, but it also leads to significant out-of-pocket costs and to productivity losses for parents or caregivers (i.e. absenteeism and presenteeism). Consequently, JIA is associated with considerable financial burden to society.

In order to analyse the cost impact of JIA, it is necessary to identify, measure and value resource use associated with the treatment or management strategies. These costs can be measured in a full economic evaluation, in which both costs and outcomes of alternative treatment or management strategies are analysed. Costs can also be measured in partial economic evaluations such as a cost description or a cost-outcome description [16]. Identifying the perspective of the analysis (ranging from health care system to full societal), determines what categories of costs are included in these analyses. As illustrated above, the impact of JIA (in terms of costs and health outcomes) cannot be fully captured by only considering the health care related costs of JIA, such as physician visits, the use of medication, or hospitalizations. Instead, it should also capture the costs of JIA borne outside of the health care sector, including productivity losses (not only for patients, but also for parents, caregivers or siblings), as well as out-of-pockets costs, such as costs for home and/or car alterations, extra school costs, transportation costs, etc. Therefore, when performing an economic evaluation of JIA, the use of a societal perspective is recommended [17, 18]. Although general guidance on conducting and reporting economic evaluations is available [19–21], these do not include a standardized list of resource use items or any standardized resource use data collection instrument to include in such evaluations. There have been efforts to standardize these resource use items but to date this is limited to adult conditions in the UK from a payer perspective [22]. In addition, two recent systematic reviews did not identify any validated standardized instruments for collecting health care resource use in children [23, 24]. Although a number of (non-validated) instruments for different childhood diseases (although not including JIA) can be found in an online database of such instruments, there is considerable variation in the resource use items they include [25]. We therefore concluded there is no standardized guidance regarding what types of resource use items to include in an economic evaluation of interventions for childhood diseases generally, nor for JIA specifically.

This study therefore aims to describe current practice on identification and measurement of health care resource use in the health economic literature on JIA. It is hypothesized that the lack of available guidance will be reflected in literature. A second objective is to identify commonly used sources to collect health care resource use and unit costs.^1^ The findings from this study will be taken into account in a multicenter, international collaborative project into management strategies for JIA, conducted in the Netherlands and Canada, named UCAN CAN-DU.^2^ The results of the current study will, ultimately, facilitate the identification of a standard set of core resource use items to improve the quality and comparability of health economic evaluations of JIA interventions.

## Methods

A scoping review was conducted to identify resource use items and unit costs included in economic evaluations or costing/resource utilization studies in JIA [26, 27]. The search was performed in PubMed and Embase in July 2018. JIA specific disease terms were combined with the search filters for economic evaluations as recommended by the Canadian Agency for Drugs and Technologies in Health (CADTH) [28] (Additional file 1). Studies were included if they met the following criteria: peer reviewed articles presenting primary research; the study type included a full or partial economic evaluation which included costs, or was an analysis of resource utilization; the study population focused on JIA patients and/or the potential consequences of JIA into adulthood; English language. As the aim of this scoping review was to capture current practice including all economic evaluation or costing/resource utilization studies that have been performed in JIA, a broadly defined search strategy was used (e.g. also including the abbreviations ‘JA’, ‘JRA’ and ‘JCA’). Unlike with a systematic review, a quality assessment was not part of the inclusion criteria for this study. Duplicates were removed electronically, and then titles and abstracts were screened by two reviewers (MMAK and GC). Related published literature reviews were excluded, but the reference lists were manually searched for relevant studies [11, 29–32]. Full text review was conducted (by MMAK and GC), and disagreements resolved by consensus. A third reviewer (LGL) was available if consensus could not be reached.

All included full-text articles were analysed (by MMAK) to identify which resource use items were measured, or which items were mentioned as (potentially) relevant (e.g. in the discussion section of the manuscript) but not measured. Subsequently, for each study, the data sources used to obtain resource use and unit costs were identified.

## Results

### Literature search

**Fig. 1:**
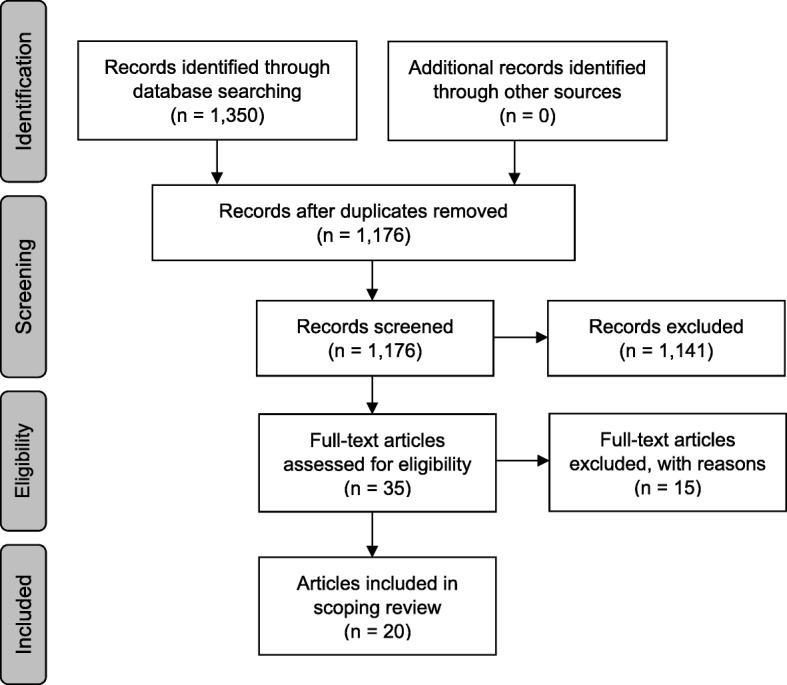
PRISMA diagram. This figure shows the results of the literature search

The search strategy retrieved 950 citations in Embase and 400 in PubMed. As expected, the broadly defined search strategy resulted in the exclusion of many irrelevant articles. Figure 1 presents an overview of the selection process, using the PRISMA reporting guidelines. This process resulted in the final inclusion of 20 full-text articles, representing 19 unique studies [9, 32–50]. As two articles reported the results of the same study it was decided to include both articles in the scoping review [47, 48]. This prevents missing any resource use or cost items, which may have occurred when not all items were mentioned in both articles. An overview of the characteristics of all included studies is provided in Table 1.

**Table 1:** Characteristics of included studies

Country	Study	Analysis type [16]^a^	Resource use included^b^	Time horizon
Canada	Bernatsky et al. (2007) [36]	Cost description	Health care Productivity School days lost	1 year
	Ens et al. (2013) [37]	Cost description	Family out-of-pocket Productivity	1 year
	Luca et al. (2016) [40]	Full economic evaluation	Health care	5 years
	Toupin et al. (2009) [35]	Resource use	Family out-of-pocket (complementary and alternative health care only)	1 year
	Ungar et al. (2011) [49]	Full economic evaluation	Health care Productivity	1 year
Finland	Haapasaari et al. (2004) [39]	Cost description	Health care Family out-of-pocket Productivity	1 year
	Pohjankoski et al. (2011) [43]	Cost-outcome description	Health care	1 year
Germany	Minden et al. (2004) [41]	Cost description	Health care Family out-of-pocket Productivity	1 year
	Minden et al. (2009) [42]	Cost description	Health care Education Family out-of-pocket Productivity School days lost	1 year
The Netherlands	Prince et al. (2011) [44]	Cost-outcome description	Health care	1 year
United Kingdom	Angelis et al. (2016) [34]	Cost-outcome description	Health care Family out-of-pocket Productivity	1 year
	Epps et al. (2005) [38]	Full economic evaluation	Health care Productivity	6 months
	Shepherd et al. (2016) [32]	Full economic evaluation	Health care	30 years
	Thornton et al. (2008.1 and 2008.2) [47, 48]	Cost description	Health care	1 year
United States	Allaire et al. (1992) [33]	Cost description	Health care Education costs Family out-of-pocket Productivity	1 year
	Rasu et al. (2015) [45]	Resource use	Productivity	9 years
	Seburg et al. (2015) [46]	Resource use	Family out-of-pocket (complementary and alternative health care only)	1 year (inferred, but not reported clearly)
Multiple countries *(Bulgaria, France, Germany, Italy, Spain, Sweden, United Kingdom)*	Kuhlmann et al. (2016) [9]	Cost-outcome description	Health care Social services Family out-of-pocket Productivity	1 year
Multiple countries *(France, Germany, the Netherlands, United Kingdom, United States)*	Shenoi et al. (2018) [50]	Cost-outcome description	Health care Education Family out-of-pocket Productivity School days lost	Assistive devices: over past 4 weeks as well from time since JIA onset. Productivity: 2 months

This table shows an overview of the studies included in the scoping review, including the country in which it was performed, the analysis type, the resource use included and the time horizon applied

^a^Studies quantifying only resource use were classified as such. All studies that describe both resource use and costs were classified according to Drummond et al, 2005 [16]

^b^The studies were categorized as including health care costs, other government agency costs (education and social services), family out-of-pocket costs, productivity costs and school time lost. These are broad categories only, and does not indicate exhaustive inclusion within the category

Of the 20 articles included, 7 articles explicitly reported the use of a societal perspective [9, 34, 38, 41, 42, 49] or a social perspective [44]. In addition, 17 articles used a time horizon of 1 year or less [9, 33–39, 41–44, 46–50], and only 4 articles conducted a full economic evaluation [32, 38, 40, 49], indicating that these studies compared costs and consequences of two or more alternatives [16].

A summary of all resource use and cost items reported in the included articles is shown in Table 2. A more detailed overview is given in Additional file 2, in which we also made note of items that were mentioned as (potentially) relevant but not actually included. As the aim of the current paper was to identify *which* resource use and cost items related to JIA were reported in literature and how they were quantified, this study does not report the magnitude of these items or the findings of the included studies.

**Table 2:** Overview of cost and resource items reported in the 20 included articles

Category	Type of cost or resource use item	Number of articles	
		N	References
Medical costs	Medication	16	[9, 32–34, 36–44, 47–49]
	DMARDs – non biologic	11	[32, 38–44, 47–49]
	*Methotrexate (tablets or subcutaneous)*	8	[32, 39, 40, 43, 44, 47–49]
	*Cyclosporin*	3	[43, 47, 48]
	*Hydroxychloroquine*	3	[43, 47, 48]
	*Sulphasalazine*	3	[43, 47, 48]
	*Leflunomide*	3	[43, 47, 48]
	*Other, or not specified*	6	[38, 41–43, 47, 48]
	DMARDs - biologic	10	[32, 38–40, 42–44, 47–49]
	*Etanercept*	6	[39, 40, 44, 47–49]
	*Abatacept*	3	[32, 40, 49]
	*Infliximab*	3	[47–49]
	*Anti-Tumor Necrosis Factor (TNF) (not further specified)*	2	[38, 43]
	*Other, or not specified*	3	[32, 42, 49]
	Corticosteroids	6	[38, 42–44, 47, 48]
	*Prednisolone*	3	[43, 47, 48]
	*Glucocorticoids (systemic)*	3	[42, 44] [43]*
	*Other, or not specified*	3	[38, 47, 48]
	NSAIDs and analgesics	8	[38, 41–44, 47, 48] [39]*
	Joint injections	8	[32, 37–39, 44, 47, 48] [43]*
	Eye drops/ointments	3	[42, 47, 48]
	Premedication	2	[40, 49]
	Osteoporosis treatment/ prophylaxis	2	[41, 42]
	Gastroprotective agents	2	[41, 42]
	Other medication, or not specified	7	[9, 33, 34, 36, 38, 47, 48]
	Outpatient and inpatient hospital visits	16	[9, 32–34, 36–42, 44, 47–50]
	Outpatient stays/day-care/visits (including joint injection, and outpatient surgery)	16	[9, 32–34, 36–42, 44, 47–50]
	Inpatient stays/inpatient treatment/surgery (including joint replacement)	13	[9, 32, 33, 37–42, 44, 47, 48, 50]
	Acute (including emergency room visits)	6	[34, 36, 37, 40–42]
	Rehabilitation	5	[9, 34, 36, 41, 42]
	Medical professional visits	14	[9, 32, 34, 36–38, 40–42, 44, 47–50]
	Rheumatology paediatric visit (or telephone consultation)	9	[32, 37, 38, 41, 42, 44, 47, 48, 50]
	Ophthalmologist	6	[32, 38, 42, 47, 48, 50]
	General practitioner visits	5	[32, 34, 36, 38, 50]
	Specialist nurse / district nurse	4	[32, 38, 47, 48]
	Nephrologist/endocrinologist	3	[38, 47, 48]
	Other, or not specified	5	[9, 38, 40, 49, 50]
	Other medical visits (including paramedical care)	13	[32, 33, 35, 37–39, 41, 42, 44, 46–48, 50]
	Physiotherapist (at health centre or at home), including hydrotherapy	11	[32, 33, 37–39, 41, 42, 44, 47, 48, 50]
	Occupational therapist	7	[32, 38, 41, 42, 47, 48, 50]
	Podiatrist/orthotics	4	[32, 38, 47, 48]
	Psychologist/counsellor	4	[38, 47, 48, 50]
	Chiropractic	2	[35, 46]
	Other, or not specified	4	[35, 38, 42, 46]
	Laboratory tests	14	[9, 32–34, 36, 38–42, 44, 47–49]
	Haemoglobin	4	[32, 38, 47, 48]
	Haematocrit	4	[32, 38, 47, 48]
	Platelets	4	[32, 38, 47, 48]
	White blood cell count	4	[32, 38, 47, 48]
	C-reactive protein (CRP)	3	[32, 47, 48]
	Liver function test	2	[32, 38]
	Tuberculosis screening	1	[49]
	Other, or not specified	12	[9, 32–34, 36, 39–42, 44, 47, 48]
	Imaging	11	[9, 32–34, 36, 38, 40, 44, 47–49]
	Radiography (X-ray)	7	[32, 33, 38, 44, 47–49]
	Magnetic resonance imaging (MRI)	5	[32, 38, 44, 47, 48]
	Ultrasound	5	[32, 38, 44, 47, 48]
	Dual-energy X-ray absorptiometry (DEXA) scan	4	[32, 44, 47, 48]
	Other, or not specified	5	[9, 34, 36, 38, 40]
	Splints and/or devices	11	[9, 33, 34, 36, 37, 40–42, 47, 48, 50]
	Orthopaedic devices (casts/splints/braces/ambulation aids wheelchair/stroller/walker frame)	8	[9, 33, 36, 37, 42, 47, 48, 50]
	Other, or not specified	5	[34, 37, 40–42]
	Supplements and/or alternative medicine (e.g. vitamins, minerals, herbal medicine)	8	[35, 38, 41, 42, 46–49]
	Folic acid	3	[47–49]
	Other, or not specified	7	[35, 38, 41, 42, 46–48]
	Drug administration costs	6	[32, 37, 39, 47–49]
	Administering joint injections (incl. appointment and/or anaesthesia)	4	[37, 39, 47, 48]
	Intravenous infusion (including bags and solutions)	3	[32, 37, 49]
	Other injections, which may include monitoring (by nurse/caregiver), and which may include training and/or caregiver time	3	[32, 39, 49]
	Other, or not specified	1	[49]
	Overhead/fixed resources	1	[38]
Out-of-pocket patient/family costs	Transportation costs	10	[9, 33, 34, 36, 37, 39, 41, 42, 44, 50]
	Transportation, non-medical (including toll)	7	[9, 33, 34, 37, 39, 41, 42]
	Transportation, medical	5	[9, 34, 37, 39, 42]
	Other, or not specified	3	[36, 44, 50]
	(Other) out-of-pocket costs	9	[33, 35, 37, 39–42, 44, 50]*
	Home adaptations and special equipment (toilet seat appliance, bathtub or shower appliance, stair lift)	5	[33, 37, 41, 42, 50]
	Childcare for babysitting, also for other children (during medical visits/hospitalization of diseased child)	4	[33, 37, 42, 50]
	Caregivers’ accommodations (e.g. when child is hospitalized/receives injection)	4	[33, 37, 50] [39]*
	Extra telephone costs	3	[33, 37, 42]
	Parking (for hospital and other medical visits)	3	[33, 37, 50]
	Money spend on food during medical visits	2	[33, 37]
	Other, or not specified	3	[35] [40, 44]*
	Social care services/home care/private and community services/ domestic help	4	[9, 34, 41, 42]
	Use of social care services (i.e. formal (paid) care) and professional caregivers, including home medical care	2	[9, 34]
	Use of private and community services/domestic help	2	[41, 42]
	School costs	3	[33, 42, 50]
Productivity costs	Productivity loss of caregivers (including informal caregiving)	14	[9, 33, 34, 36–42, 44, 45, 49, 50]*
	Work/sick leave due to child’s illness, time lost due to health care appointments and/or due to informal caregiving (absenteeism)	12	[9, 33, 34, 36–39, 42, 45, 49, 50] [41]*
	Cease employment/early retirement	3	[9, 34, 50]
	Other, or not specified (including reduced number of working hours, and presenteeism)	3	[50] [40, 44]*
	Missed school days and productivity loss of patients	8	[9, 33, 34, 36, 40–42, 50]*
	Sick leave from school	4	[36, 42, 50] [41]*
	Sick leave from work	3	[9, 34, 41]
	Cease employment/early retirement	3	[9, 34, 41]
	Impact on future employment ability	3	[33, 36, 40]*
	Missed school days of siblings	1	[50]

This table shows the costs or resource use items that are either measured in the articles from the scoping review or mentioned as relevant items. A further specification of these items was shown in the second column, and the number of articles and accompanying references in which these items were measured are shown in columns three and four. In case items were only mentioned (e.g. in the study’s discussion section), this is indicated with an asterisk (*). Items that were only included in one study or not specified in detail, were summarized into the category ‘Other, or not specified’

### Reporting of resource use and cost items

As shown in Table 2, when considering medical costs, medication use was reported in 16 out of 20 articles. The remaining articles had a narrow focus (i.e. only included productivity losses, or only focused on alternative and complementary medicine), which explains why medication use was not reported. Of the 20 articles, outpatient and inpatient hospital visits were reported in 16 articles (e.g. including joint injections, day-care admissions and hospitalizations), medical professional visits in 14 articles (e.g. paediatric rheumatologists or other physician visits), and 13 articles reported other medical visits (including paramedical care). Laboratory tests were reported in 14 articles, and imaging in 11 articles. Other health care related items that were reported involved splints and/or devices (11 articles), the use of supplements/alternative medicine (8 articles), drug administration (6 articles) and hospital overhead (i.e. heating, lighting and administration) (1 article).

Of the 8 articles that reported the use of supplements or alternative medicine, 2 articles specifically investigated the use of complementary and/or alternative medicine, and did not estimate the accompanying impact on costs [35, 46]. In these studies, the percentage of patients using complementary and alternative health care was found to range between 36% (within a one-year period) [35] and 72% (without specifying a time horizon) [46].

With regard to out-of-pocket payments and (other) family costs, transportation costs were found to be reported in 10 articles, (other) out-of-pocket costs in 9 articles, social care services and/or home care in 4 articles, and (additional) school costs in 3 articles.

Productivity losses by *parents and/or caregivers* were mentioned in 14 articles, although only 11 actually included it in their evaluation. The remaining 3 articles did mention parents’ productivity losses but did not aim to include it in their evaluation [40, 41], or could not include it because parents were reluctant to fill out cost diaries [44]. In contrast, when considering the *child* with JIA, missed school days or productivity losses were mentioned in 8 articles, but only included in 6 articles. In addition, 1 article (also) reported the impact on siblings [50].

Although 4 articles included or mentioned adverse events, side effects and/or complications due to the disease and/or treatment [32, 36, 40, 50], these were not included as a separate category as these involve hospital visits, treatment, and other items already included in Table 2. Similarly, costs of JIA over the long term were included or mentioned in 8 articles [32, 34, 36, 37, 40, 47–49], but (whenever possible) the accompanying resource use items (e.g. surgery and treatment) were captured in the other items.

### Data sources used to obtain resource use and unit costs

The main data sources or data collection instruments where *resource use* was obtained from are: questionnaires or inquiry with parent(s), caregivers or child; chart review, medical records, or patient files; published or accessible data(bases) or literature; physician inquiry or expert opinion; and (case) report forms or other data collection methods (Table 3).

**Table 3:** Overview of data sources used to obtain resource use

Category	Type of resource use item	Total	Source from which resource use was obtained [N (references)]						
		(N [references])	Questionnaire / inquiry with parent(s) or caregivers / child; recall	Chart review / medical records / patient files	Published / available data(base) or literature	Physician inquiry / caregiver inquiry/ expert opinion	(Case) report forms / collected during study (intervention)	From hospital	From health insurer
Medical costs	Medication	16 [9, 32–34, 36–44, 47–49]	8 [9, 34, 36–39, 41, 42]	5 [33, 37, 39, 41, 44]	5 [32, 40, 47–49]	4 [42, 44, 47, 48]	2 [47, 48]		1 [43]
	Outpatient and inpatient hospital visits	16 [9, 32–34, 36–42, 44, 47–50]	9 [9, 34, 36–39, 41, 42, 50]	5 [33, 37, 39, 41, 44]	5 [32, 40, 47–49]	1 [44]	3 [44, 47, 48]		
	Medical professional visits	14 [9, 32, 34, 36–38, 40–42, 44, 47–50]	8 [9, 34, 36–38, 41, 42, 50]	3 [37, 41, 44]	5 [32, 40, 47–49]	4 [38, 44, 47, 48]	3 [38, 47, 48]		
	Other medical visits (including paramedical care)	12 [32, 33, 37–39, 41, 42, 44, 46, 47, 48, 50]	9 [33, 35, 37–39, 41, 42, 46, 50]	3 [39, 41, 44]	3 [32, 47, 48]	4 [38, 44, 47, 48]	3 [38, 47, 48]		
	Laboratory tests	14 [9, 32–34, 36, 38–42, 44, 47–49]	6 [9, 34, 36, 38, 39, 41]	4 [33, 39, 41, 44]	5 [32, 40, 47–49]	2 [42, 44]	2 [47, 48]		
	Imaging	11 [9, 32–34, 36, 38, 40, 44, 47–49]	4 [9, 34, 36, 38]	1 [33]	5 [32, 40, 47–49]	1 [44]	3 [44, 47, 48]		
	Splints and/or devices	11 [9, 33, 34, 36, 37, 40–42, 47, 48, 50]	10 [9, 33, 34, 36, 37, 41, 42, 47, 48, 50]		1 [40]				
	Supplements and/or alternative medicine (e.g. vitamins, minerals, herbal medicine)	8 [35, 38, 41, 42, 46–49]	5 [35, 38, 41, 42, 46]	1 [41]	3 [47–49]	3 [42, 47, 48]	2 [47, 48]		
	Drug administration costs	6 [32, 37, 39, 47–49]	1 [39]	2 [37, 39]	4 [32, 47–49]		2 [47, 48]		
	Overhead/fixed resources	1 [38]						1 [38]	
Out-of-pocket patient/family costs	Transportation costs	10 [9, 33, 34, 36, 37, 39, 41, 42, 44, 50]	10 [9, 33, 34, 36, 37, 39, 41, 42, 44, 50]						
	(Other) out-of-pocket costs	7 [33, 35, 37, 41, 42, 44, 50]	7 [33, 35, 37, 41, 42, 44, 50]						
	Social care services/home care/ private and community services	4 [9, 34, 41, 42]	3 [9, 41, 42]			1 [34]			
	School costs	3 [33, 42, 50]	3 [33, 42, 50]						
Productivity costs	Productivity loss of caregivers (including informal caregiving)	12 [9, 33, 34, 36–39, 42, 44, 45, 49, 50]^a^	10 [9, 33, 34, 36–39, 42, 44, 50]		2 [45, 49]				
	Missed school days and productivity loss of patients	6 [9, 34, 36, 41, 42, 50]	5 [9, 34, 36, 42, 50]	1 [41]					
	Missed school days of siblings	1 [50]	1 [50]						

This table shows the resource items that are included in the 20 articles from this scoping review, and the data sources that are used to obtain this resource use. The number of articles for each of the different data sources, and the accompanying references, are provided

^a^Prince et al. (2011) [44] did provide information on which data source they intended to use to collect productivity losses among caregivers, but they were not able to include it in the final evaluation due to lack of available data

When considering the data sources used to obtain *unit costs*, the results indicate that these are most commonly based on: medical charges or reimbursement fees; published or available data(bases), literature, or guidelines; hospital-based fees or costs; and questionnaire or inquiry with the parent(s) or the child (Table 4). The impact of JIA on missed school days could not be expressed in monetary units.

**Table 4:** Overview of data sources used to obtain costs (unit costs)

Category	Type of cost item	Total	Unit costs obtained from [N (references)]						
		(N [references])	Medical charges / payment by results database / (average) reimbursement fee	Published / available data(base) or literature / guidelines	Hospital-based fees / costs	Questionnaire / inquiry with parent(s) / child; recall	Provided by manufacturer / supplier (e.g. pharmacist)	Assumption / not specified	Not costed
Medical costs	Medication	16 [9, 32–34, 36–44, 47–49]	8 [9, 32, 33, 36, 40, 41, 43, 49]	8 [9, 32, 34, 38, 42, 44, 47, 48]	1 [38]	2 [33, 37]	1 [39]		
	Outpatient and inpatient hospital visits	15 [9, 32–34, 36–42, 44, 47–49]	6 [9, 32–34, 41, 42]	10 [32, 34, 36, 38, 40–42, 47–49]	5 [38, 39, 44, 47, 48]	2 [33, 37]			
	Medical professional visits and care	13 [9, 32, 34, 36–38, 40–42, 44, 47–49]	9 [9, 32, 34, 36, 41, 42, 47–49]	8 [9, 32, 34, 38, 40, 41, 47, 48]	4 [38, 44, 47, 48]	1 [37]			
	Other medical visits (including paramedical care)	10 [32, 33, 37–39, 41, 42, 44, 47, 48]	6 [32, 33, 41, 42, 47, 48]	5 [32, 38, 41, 47, 48]	5 [38, 39, 44, 47, 48]	2 [33, 37]			
	Laboratory tests	14 [9, 32–34, 36, 38–42, 44, 47–49]	9 [9, 32–34, 36, 40, 47–49]	6 [32, 34, 38, 41, 42, 49]	4 [36, 38, 39, 44]	1 [33]			
	Imaging	11 [9, 32–34, 36, 38, 40, 44, 47–49]	8 [9, 32–34, 36, 38, 47, 48]	4 [32, 34, 38, 49]	4 [40, 44, 47, 48]	1 [33]			
	Splints and/or devices	10 [9, 33, 34, 36, 37, 40–42, 47, 48]	4 [33, 36, 40, 41]	2 [47, 48]		2 [33, 37]	3 [9, 34, 36]	1 [42]	
	Supplements and/or alternative medicine (e.g. vitamins, minerals, herbal medicine)	8 [35, 38, 41, 42, 46–49]	4 [35, 41, 46, 49]	4 [38, 42, 47, 48]	1 [38]				
	Drug administration costs	6 [32, 37, 39, 47–49]		3 [32, 47, 48]	1 [49]	1 [37]		1 [39]	
	Overhead/fixed resources	1 [38]			1 [38]				
Out-of-pocket patient/family costs	Transportation costs	10 [9, 33, 34, 36, 37, 39, 41, 42, 44, 50]	2 [9, 39]	1 [36]		8 [33, 34, 36, 37, 41, 42, 44, 50]			
	(Other) out-of-pocket costs	6 [33, 37, 41, 42, 44, 50]				6 [33, 37, 41, 42, 44, 50]			
	Social care services/ home care/ private and community services/ domestic help	4 [9, 34, 41, 42]	1 [9]	1 [34]		2 [41, 42]			
	School costs	2 [33, 42]				2 [33, 42]			
Productivity costs	Productivity loss of caregivers (including informal caregiving)	10 [9, 33, 34, 36–39, 42, 45, 49]		8 [9, 34, 37–39, 42, 45, 49]		3 [33, 36, 37]			
	Missed school days and productivity loss of patients	5 [9, 34, 36, 41, 42]		4 [9, 34, 41, 42]					2 [36, 41]
	Missed school days of siblings	0							

This table shows the unit costs that are included in the 20 articles from this scoping review, and the data sources that are used to obtain these. The number of articles for each of the different data sources, and the accompanying references, are provided

## Discussion

Medication use, outpatient and inpatient hospital visits, medical professional, other medical visits, and laboratory tests were found to be the most commonly reported health care related resource use items. Besides these, when considering resource use items related to costs of lost productivity and costs borne by families, productivity losses among parents and/or caregivers were most frequently reported. However, the extent and way these are incorporated differs considerably between studies. Other resource use items to quantify impacts outside the health care system, including social care services, school costs, school days lost and productivity losses among patients, as well as transportation costs and (other) out-of-pocket costs, were less frequently reported.

The most commonly reported resource use items (i.e. medication, medical professional and other medical visits, outpatient and inpatient hospital visits) mirror the core items identified for an adult generic health care resource use instrument [51], with the addition of laboratory testing. The addition of laboratory testing in the JIA population aligns with clinical practice, given that the medications prescribed require regular lab monitoring (both in adults and children). In addition, the relatively common inclusion of productivity losses of caregivers is reflective of this unique consideration in the evaluation of childhood diseases.

When considering the 20 articles included, there is strong variation in the perspectives (or combination of perspectives) that were applied. In addition, the great majority of these studies used a one-year time horizon, although this is insufficient to capture the full economic implications of childhood diseases like JIA. The choice for these different perspectives and time horizons may however be partly explained by the different aims of the articles, ranging from (specifically) investigating out-of-pocket costs and the use of complementary or alternative health care [35, 37, 46], to investigating a specific intervention or type of medication [38, 44, 49]. Therefore, the high heterogeneity between the articles in this scoping review limits their comparability. In addition, as studies are performed in different countries, with different health care systems, the resource use and cost items covered may differ between these health care systems, thereby further limiting the comparability between the studies in this scoping review. Consequently, the full health economic impact of JIA cannot be reliably quantified.

Although questionnaires or inquiry with parent(s) or the child were found to be the most frequently used data source to obtain resource use, the impact of JIA on societal costs (including lost productivity) is often not incorporated. More specifically, the impact of JIA in terms of missed school days was only incorporated in 3 articles (although none of them quantified this impact in terms of costs), making it likely that many health economic evaluations underestimated the full potential impact of JIA.

Although medical records will likely represent the most reliable source of evidence for collecting medical resource use (in contrast to questionnaires or inquiry with parents), results indicate that this is not common practice. As results from questionnaires are prone to recall bias, the accuracy of the outcomes of these studies may be limited. When considering unit costs, the results indicate that these were most frequently obtained from medical charges or reimbursement fees, or from published or available data(bases), literature, or costing guidelines. Although medical charges and reimbursement fees may not provide the actual costs incurred by the health service provided, they are however often considered the best available source of evidence.

This is the first study to provide a comprehensive overview of all resource use items included in studies evaluating JIA-related resource use and/or costs. Although some resource use items identified (e.g. splints and devices) may be specific to JIA, most items will also apply to other (chronic) childhood diseases. However, as mentioned previously, no validated health care resource data collection instrument for children could be found. Therefore, the results from the current study, in terms of the importance of improved guidance regarding how to quantify resource use and unit costs, are likely also valuable to other (chronic) childhood diseases.

The results of this scoping review are limited by the quality of reporting on the included resource use items. One limitation concerns the level of detail reported for each item. For example, although medical professional visits were included in most of the articles, the type of medical professional that was consulted was not always stated explicitly. This lack of detail limits the transparency and reproducibility of the study’s results.

Another limitation concerns the accuracy in reporting data sources for quantifying resource use and obtaining unit costs. This specification is often general, for example stating that all resource use was obtained from physician inquiry and/or available literature, or stating that all medical costs were obtained from medical charges and/or published literature, without exactly specifying which data source was used.

In health economic evaluations, the use of a wider and/or societal perspective is increasingly recommended as the preferred perspective, and is required when it is likely that this wider and/or societal impact will substantially impact the results [17, 18, 52]. Thus, as JIA leads to productivity losses among both patients and parents and/or caregivers [9, 34, 36] and comes with significant costs borne by families, it can be concluded that this impact is often overlooked as only 7 out of 20 articles report the use of a societal (or social) perspective. More specifically, when considering the resource use and costs that were actually included in these 7 articles, this varies from only incorporating health care costs and productivity losses (although use of the term societal perspective should not be justifiable purely based on the inclusion of productivity losses [53]), to also incorporating out-of-pocket costs, lost school days, social costs, and/or costs of education. Thus, there is great variation in the way the societal perspective is conceptualized and interpreted in economic evaluations, which has also been reported in literature [53, 54].

Another aspect that may be overlooked in economic evaluations of JIA concerns the use of complementary and alternative health care. As the results of the current study show that this type of health care is used by a substantial proportion of JIA patients, incorporating the accompanying costs is likely relevant to capture out-of-pocket costs for families. Consequently, current economic evaluations of JIA (as well as other (chronic) childhood diseases) may underestimate the real-life impact of JIA.

In addition, the terminology used to state the perspective of the evaluation was not always clear. To illustrate this, 2 articles that reported either the use of a ‘direct medical cost perspective’ [36] or a ‘direct and indirect cost perspective’ [39] both reported (besides medical costs) working time lost from parents or caregivers and/or family borne costs. Consequently, the quality and comparability of studies evaluating resource use and costs in JIA would benefit from improved guidance regarding: 1) which perspective can and should be applied, 2) which cost and resource use items should be included, and 3) how these items can be quantified. Such improved guidance will likely increase the quality and comparability between these studies [51], both for JIA as well as for other chronic childhood diseases.

## Conclusions

Although there is some consistency in terms of items that are included in studies examining resource use and unit costs of JIA from the health care system perspective, there is heterogeneity in what is included within perspectives going beyond that. However, as the impact of productivity losses and family borne costs among JIA patients (and among other chronic childhood diseases) pose a substantial burden to society, these studies should go beyond the health care system perspective to capture this full societal impact. Therefore, improved guidance for conducting and reporting (health) economic evaluations of JIA is required. This can be achieved by the development of a standardized list of items for collecting resource use and unit costs in chronic childhood diseases. In particular it should be emphasized how to quantify this societal impact of these childhood diseases over a prolonged time-period. Incorporating these recommendations will likely increase the quality and comparability of health economic evaluations of both JIA and other chronic childhood diseases.

## Additional files

Additional file 1: Overview of search strategy. This file provides an overview of the search strategy performed in Embase and in PubMed. (DOCX 17 kb)

Additional file 2: Results of scoping review. This file provides an extensive overview of the results of the scoping review. (DOCX 564 kb)

## Abbreviations

CADTH: Canadian Agency for Drugs and Technologies in Health
DIRUM: Database of Instruments for Resource Use Measurement
DMARD: Disease-modifying antirheumatic drug
HRQoL: Health-related quality of life
JIA: Juvenile idiopathic arthritis
MTX: Methotrexate
NSAIDs: Non-steroidal anti-inflammatory drugs
UCAN CAN-DU: Canada-Netherlands Personalized Medicine Network in Childhood Arthritis and Rheumatic diseases

## Footnotes

1 The term ‘unit costs’ indicates the mean costs per unit. The total costs per resource use item can be calculated by multiplying the unit costs with the accompanying resource utilization.

2 UCAN CAN-DU is the Canada-Netherlands Personalized Medicine Network in Childhood Arthritis and Rheumatic diseases.
