# Supplementary material for: Seeking the state of the art in standardized measurement of health care resource use and costs in juvenile idiopathic arthritis: a scoping review
Source: Pediatr Rheumatol Online J. 2019 May 6;17:20. doi: 10.1186/s12969-019-0321-x (PMC6501309; doi:10.1186/s12969-019-0321-x)
Supplement: Supplementary file 1 — Overview of search strategy. This file provides an overview of the search strategy performed in Embase and in PubMed. (DOCX 17 kb) [file 12969_2019_321_MOESM1_ESM.docx]

**Additional file 1: Overview of search strategy**

**Search strategy performed in Embase (based on the search filters recommended by the CADTH (1)):**

1. economics/

2. Cost/

3. exp Health Economics/

4. Budget/

5. budget*.ti,ab,kw.

6. (economic* or cost or costs or costly or costing or price or prices or pricing or pharmacoeconomic* or pharmaco-economic* or expenditure or expenditures or expense or expenses or financial or finance or finances or financed).ti,kw.

7. (economic* or cost or costs or costly or costing or price or prices or pricing or pharmacoeconomic* or pharmaco-economic* or expenditure or expenditures or expense or expenses or financial or finance or finances or financed).ab. /freq=2

8. (cost* adj2 (effective* or utilit* or benefit* or minimi* or analy* or outcome or outcomes)).ab,kw.

9. (value adj2 (money or monetary)).ti,ab,kw.

10. Statistical Model/

11. economic model*.ab,kw.

12. Probability/

13. markov.ti,ab,kw.

14. monte carlo method/

15. monte carlo.ti,ab,kw.

16. Decision Theory/

17. Decision Tree/

18. (decision* adj2 (tree* or analy* or model*)).ti,ab,kw.

19. or/1-18

20. juvenile rheumatoid arthritis/

21. juvenile idiopathic arthritis.ti,ab,kw.

22. pediatric rheumatic disease.ti,ab,kw.

23. JIA.ti,ab,kw.

24. ja.ti,ab,kw.

25. jra.ti,ab,kw.

26. jca.ti,ab,kw.

27. childhood arthritis.ti,ab,kw.

28. juvenile rheumatoid arthritis.ti,ab,kw.

29. juvenile chronic arthritis.ti,ab,kw.

30. or/20-29

31. 19 and 30

**Search strategy performed in PubMed (based on the search filters recommended by the CADTH (1)):**

Search (((Economics[Mesh:NoExp] OR "Costs and Cost Analysis"[mh] OR Economics, Nursing[mh] OR Economics, Medical[mh] OR Economics, Pharmaceutical[mh] OR Economics, Hospital[mh] OR Economics, Dental[mh] OR "Fees and Charges"[mh] OR Budgets[mh] OR budget*[tiab] OR economic*[tiab] OR cost[tiab] OR costs[tiab] OR costly[tiab] OR costing[tiab] OR price[tiab] OR prices[tiab] OR pricing[tiab] OR pharmacoeconomic*[tiab] OR pharmaco-economic*[tiab] OR expenditure[tiab] OR expenditures[tiab] OR expense[tiab] OR expenses[tiab] OR financial[tiab] OR finance[tiab] OR finances[tiab] OR financed[tiab] OR value for money[tiab] OR monetary value*[tiab] OR models, economic[mh] OR economic model*[tiab] OR markov chains[mh] OR markov[tiab] OR monte carlo method[mh] OR monte carlo[tiab] OR Decision Theory[mh] OR decision tree*[tiab] OR decision analy*[tiab] OR decision model*[tiab]) AND ((juvenile idiopathic arthritides[MeSH Terms]) OR (juvenile idiopathic arthritis[MeSH Terms]) OR (juvenile chronic arthritis[MeSH Terms]) OR (juvenile rheumatoid arthritis[Title/Abstract]) OR (juvenile rheumatoid arthritis[Title/Abstract]) OR (juvenile idiopathic arthritis[Title/Abstract]) OR (pediatric rheumatic disease[Title/Abstract]) OR (paediatric rheumatic disease[Title/Abstract]) OR (JIA[Title/Abstract]) OR (JA[Title/Abstract]) OR (JRA[Title/Abstract]) OR (childhood arthritis[Title/Abstract]) OR (juvenile chronic arthritis[Title/Abstract]) OR (JCA[Title/Abstract])))

**References:**

1. CADTH. Strings attached: CADTH's Database Search Filters. 2016 [cited 2017 July 15]; Available from: <https://www.cadth.ca/resources/finding-evidence/strings-attached-cadths-database-search-filters>
