# Supplementary material for: Seeking the state of the art in standardized measurement of health care resource use and costs in juvenile idiopathic arthritis: a scoping review
Source: Pediatr Rheumatol Online J. 2019 May 6;17:20. doi: 10.1186/s12969-019-0321-x (PMC6501309; doi:10.1186/s12969-019-0321-x)
Supplement: Supplementary file 2 — Results of scoping review. This file provides an extensive overview of the results of the scoping review. (DOCX 564 kb) [file 12969_2019_321_MOESM2_ESM.docx]

**Additional file 2: results of scoping review**

***Table A1.*** *Overview of cost and resource items. The first column shows the type of costs or resource use that is either measured in the articles from the scoping review, or mentioned as relevant item to be included. In articles where these items were further specified, this specification was shown in the second column, and the number of articles and accompanying references in which these items were measured (or mentioned) are shown for each item. All items that were not specified in detail, were summarized into the category ‘Other, or not specified’. Although 3 articles reported adverse events and/or complications (1-3), these were not included as a separate category as these involve hospitalizations, treatment, and other items already included in the table. Similarly, long-term care costs were included in 1 study (4), and ‘mentioned’ in 5 articles (1-3, 5, 6), but the accompanying resource use (e.g. surgery and treatment) would already be captured in the other items in this list.*

|  | **Type of cost or resource use item** | | | **Number of articles and references** | | | |
| --- | --- | --- | --- | --- | --- | --- | --- |
|  |  |  |  | **Included** | | **Mentioned** | |
|  |  |  |  | **N** | **References** | **N** | **References** |
| Medical costs | Medication | | | 16 | (1-16) | - |  |
|  |  | DMARDs - non biologic | | 11 | (1, 3, 4, 8, 9, 11-16) | - |  |
|  |  |  | Methotrexate (tablets or subcutaneous) | 8 | (1, 3, 4, 9, 13-16) | - |  |
|  |  |  | Cyclosporin | 3 | (13, 15, 16) | - |  |
|  |  |  | Hydroxychloroquine | 3 | (13, 15, 16) | - |  |
|  |  |  | Sulphasalazine | 3 | (13, 15, 16) | - |  |
|  |  |  | Leflunomide | 3 | (13, 15, 16) | - |  |
|  |  |  | Naproxen | 2 | (15, 16) | - |  |
|  |  |  | Gold sodium thiomalate | 1 | (13) | - |  |
|  |  |  | Auranofin | 1 | (13) | - |  |
|  |  |  | Azathioprine | 1 | (13) | - |  |
|  |  |  | Podophyllotoxin | 1 | (13) | - |  |
|  |  |  | Not specified | 3 | (8, 11, 12) | - |  |
|  |  | DMARDs - biologic | | 10 | (1, 3, 4, 8, 9, 12-16) | - |  |
|  |  |  | Etanercept | 6 | (1, 4, 9, 14-16) | - |  |
|  |  |  | Abatacept | 3 | (1, 3, 4) | - |  |
|  |  |  | Infliximab | 3 | (4, 15, 16) | - |  |
|  |  |  | Anti-Tumor Necrosis Factor (TNF) (not further specified) | 2 | (8, 13) | - |  |
|  |  |  | Adalimumab | 1 | (4) | - |  |
|  |  |  | Tocilizumab | 1 | (3) | - |  |
|  |  |  | Not specified | 1 | (12) | - |  |
|  |  | Corticosteroids | | 6 | (8, 12-16) | - |  |
|  |  |  | Prednisolone | 3 | (13, 15, 16) | - |  |
|  |  |  | Glucocorticoids (systemic) | 2 | (12, 14) | 1 | (13) |
|  |  |  | Methylprednisolone | 2 | (15, 16) | - |  |
|  |  |  | Not specified | 1 | (8) | - |  |
|  |  | NSAIDs and analgesics | | 7 | (8, 11-16) | 1 | (9) |
|  |  |  | Celecoxib | 2 | (15, 16) | - |  |
|  |  |  | Codeine | 2 | (15, 16) | - |  |
|  |  |  | Diclofenac | 2 | (15, 16) | - |  |
|  |  |  | Etoricoxib | 2 | (15, 16) | - |  |
|  |  |  | Ibuprofen | 2 | (15, 16) | - |  |
|  |  |  | Ibuprofen retard | 2 | (15, 16) | - |  |
|  |  |  | Indomethacin | 2 | (15, 16) | - |  |
|  |  |  | Paracetamol | 2 | (15, 16) | - |  |
|  |  |  | Piroxicam | 2 | (15, 16) | - |  |
|  |  |  | Rofecoxib | 2 | (15, 16) | - |  |
|  |  |  | Not specified | 5 | (8, 11-14) | 1 | (9) |
|  |  | Joint injections | | 7 | (3, 5, 8, 9, 14-16) | 1 | (13) |
|  |  |  | Depomedrone | 2 | (15, 16) | - |  |
|  |  |  | Triamcinolone acetonide | 2 | (15, 16) | - |  |
|  |  |  | Not specified | 5 | (3, 5, 8, 9, 14) | 1 | (13) |
|  |  | Eye drops/ointments | | 3 | (12, 15, 16) | - |  |
|  |  |  | Atropine | 2 | (15, 16) | - |  |
|  |  |  | Betamethasone | 2 | (15, 16) | - |  |
|  |  |  | Cyclopentolate | 2 | (15, 16) | - |  |
|  |  |  | Dexamethasone | 2 | (15, 16) | - |  |
|  |  |  | Prednisolone | 2 | (15, 16) | - |  |
|  |  |  | Prednisolone forte | 2 | (15, 16) | - |  |
|  |  |  | Not specified | 1 | (12) | - |  |
|  |  | Premedication | | 2 | (1, 4) | - |  |
|  |  |  | Acetaminophen | 1 | (4) | - |  |
|  |  |  | Diphenhydramine | 1 | (4) | - |  |
|  |  |  | Hydrocortisone | 1 | (4) | - |  |
|  |  |  | Not specified | 1 | (1) | - |  |
|  |  | Antibiotics (not specified) | | 1 | (8) | - |  |
|  |  | Osteoporosis treatment/ prophylaxis | | 2 | (11, 12) | - |  |
|  |  | Gastroprotective agents | | 2 | (11, 12) | - |  |
|  |  | Medication – other | | 2 | (15, 16) | - |  |
|  |  |  | Domperidone | 2 | (15, 16) | - |  |
|  |  |  | Lansoprazole | 2 | (15, 16) | - |  |
|  |  |  | Omeprazole | 2 | (15, 16) | - |  |
|  |  |  | Ondansentron | 2 | (15, 16) | - |  |
|  |  |  | Ranitidine | 2 | (15, 16) | - |  |
|  |  |  | Pamidronate | 2 | (15, 16) | - |  |
|  |  | Other, or not specified | | 6 | (2, 6, 7, 10, 15, 16) | - |  |
|  | Outpatient and inpatient hospital visits | | | 16 | (1-12, 14-17) | - |  |
|  |  | | Outpatient stays/daycare/visits (including joint injection, and outpatient surgery) | 16 | (1-12, 14-17) | - |  |
|  |  |  | Inpatient stays/inpatient treatment/surgery (including joint replacement) | 13 | (1, 3, 5, 7-12, 14-17) | - |  |
|  |  |  | Acute (including emergency room visits) | 6 | (1, 2, 5, 6, 11, 12) | - |  |
|  |  |  | Rehabilitation | 5 | (2, 6, 10-12) | - |  |
|  | Medical professional visits | | | 14 | (1-6, 8, 10-12, 14-17) |  |  |
|  |  | | Rheumatology pediatric visit (or email or telephone consultation) | 9 | (3, 5, 8, 11, 12, 14-17) | - |  |
|  |  |  | Ophthalmologist | 6 | (3, 8, 12, 15-17) | - |  |
|  |  |  | General practitioner visits | 5 | (2, 3, 6, 8, 17) | - |  |
|  |  |  | Specialist nurse / district nurse | 4 | (3, 8, 15, 16) | - |  |
|  |  |  | Nephrologist/endocrinologist | 3 | (8, 15, 16) | - |  |
|  |  |  | Cardiologist | 2 | (15, 16) | - |  |
|  |  |  | Dermatologist | 2 | (15, 16) | - |  |
|  |  |  | Ear/nose/throat physician | 1 | (8) | - |  |
|  |  |  | Orthodontist | 1 | (8) | - |  |
|  |  |  | Orthopedic surgeon | 1 | (8) | - |  |
|  |  |  | Hematologist | 1 | (8) | - |  |
|  |  |  | Radiologist | 1 | (8) | - |  |
|  |  |  | Pediatrician | 1 | (17) | - |  |
|  |  |  | Not specified | 3 | (1, 4, 10) | - |  |
|  | Other medical visits (including paramedical care) | | | 13 | (3, 5, 7-9, 11, 12, 14-19) | - |  |
|  |  | | Physiotherapist (at health center or at home), including hydrotherapy | 11 | (3, 5, 7-9, 11, 12, 14-17) | - |  |
|  |  |  | Occupational therapist | 7 | (3, 8, 11, 12, 15-17) | - |  |
|  |  |  | Podiatrist/orthotics | 4 | (3, 8, 15, 16) | - |  |
|  |  |  | Psychologist/counsellor | 4 | (8, 15-17) | - |  |
|  |  |  | Chiropractic | 2 | (18, 19) | - |  |
|  |  |  | Massage | 2 | (18, 19) | - |  |
|  |  |  | Social worker, patient/parent education | 2 | (8, 12) | - |  |
|  |  |  | Dietician | 2 | (15, 16) | - |  |
|  |  |  | Social worker | 1 | (8) | - |  |
|  |  |  | Patient/parent education | 1 | (12) | - |  |
|  |  |  | Osteopathy | 1 | (18) | - |  |
|  |  |  | Homeopathy | 1 | (18) | - |  |
|  |  |  | Naturopathy | 1 | (18) | - |  |
|  |  |  | Hypnosis | 1 | (18) | - |  |
|  |  |  | Reflexology | 1 | (18) | - |  |
|  |  |  | Spiritual healing | 1 | (18) | - |  |
|  |  |  | Mind-body therapies (yoga, meditation, guided imagery, relaxation) | 1 | (19) | - |  |
|  | Laboratory tests | | | 14 | (1-4, 6-12, 14-16) | - |  |
|  |  | | Hemoglobin | 4 | (3, 8, 15, 16) | - |  |
|  |  |  | Hematocrit | 4 | (3, 8, 15, 16) | - |  |
|  |  |  | Platelets | 4 | (3, 8, 15, 16) | - |  |
|  |  |  | White blood cell count | 4 | (3, 8, 15, 16) | - |  |
|  |  |  | C-reactive protein (CRP) | 3 | (3, 15, 16) | - |  |
|  |  |  | Liver function test | 2 | (3, 8) | - |  |
|  |  |  | Lymphocyte count | 2 | (15, 16) | - |  |
|  |  |  | Neutrophil count | 2 | (15, 16) | - |  |
|  |  |  | Erythrocyte sedimentation ratio (ESR) | 2 | (15, 16) | - |  |
|  |  |  | Rheumatoid factor | 2 | (15, 16) | - |  |
|  |  |  | Human leukocyte antigen-B27 (HLA-B27) | 2 | (15, 16) | - |  |
|  |  |  | Immunoglobulins | 2 | (15, 16) | - |  |
|  |  |  | Tuberculosis screening | 1 | (4) | - |  |
|  |  |  | Urea | 1 | (3) | - |  |
|  |  |  | Electrolytes | 1 | (3) | - |  |
|  |  |  | Not specified | 9 | (1, 2, 6, 7, 9-12, 14) | - |  |
|  | Imaging | | | 11 | (1-4, 6-8, 10, 14-16) |  |  |
|  |  | | Radiography (X-ray) | 7 | (3, 4, 7, 8, 14-16) | - |  |
|  |  |  | Magnetic resonance imaging (MRI) | 5 | (3, 8, 14-16) | - |  |
|  |  |  | Ultrasound | 5 | (3, 8, 14-16) | - |  |
|  |  |  | Dual-energy X-ray absorptiometry (DEXA) scan | 4 | (3, 14-16) | - |  |
|  |  |  | Electrocardiogram (ECG) | 1 | (8) | - |  |
|  |  |  | Barium meal | 1 | (8) | - |  |
|  |  |  | Gastroscopy | 1 | (8) | - |  |
|  |  |  | Not specified | 4 | (1, 2, 6, 10) | - |  |
|  | Splints and/or devices | | | 11 | (1, 2, 5-7, 10-12, 15-17) | - |  |
|  |  | | Orthopedic devices (casts/splints/braces/ambulation aids/wheelchair/stroller/walker frame) | 8 | (2, 5, 7, 10, 12, 15-17) | - |  |
|  |  |  | Not specified | 5 | (1, 5, 6, 11, 12) | - |  |
|  | Supplements/alternative medicine (e.g. vitamins, minerals, herbal medicine) | | | 8 | (4, 8, 11, 12, 15, 16, 18, 19) | - |  |
|  |  | | Folic acid | 3 | (4, 15, 16) | - |  |
|  |  |  | Calcium | 2 | (15, 16) | - |  |
|  |  |  | Ferrous sulfate | 2 | (15, 16) | - |  |
|  |  |  | Sodium feredetate | 2 | (15, 16) | - |  |
|  |  |  | Cod liver oil | 1 | (8) | - |  |
|  |  |  | Aromatherapy | 1 | (8) | - |  |
|  |  |  | Not specified | 4 | (11, 12, 18, 19) | - |  |
|  | Drug administration costs | | | 6 | (3-5, 9, 15, 16) | - |  |
|  |  | | Administering joint injections (incl. appointment and/or anesthesia) | 4 | (5, 9, 15, 16) | - |  |
|  |  |  | Intravenous infusion (including bags and solutions) | 3 | (3-5) | - |  |
|  |  |  | Other injections, which may include monitoring (by nurse/caregiver), and which may include training and/or caregiver time | 3 | (3, 4, 9) | - |  |
|  |  |  | Dispensing fees/pharmacy preparation | 1 | (4) | - |  |
|  | Overhead/fixed resources | | | 1 | (8) | - |  |
|  |  | | Heating and lighting | 1 | (8) | - |  |
|  |  |  | Administration | 1 | (8) | - |  |
|  |  |  | Maintenance costs | 1 | (8) | - |  |
|  |  |  | Running costs | 1 | (8) | - |  |
| Out-of-pocket patient/family costs | Transportation costs | | | 10 | (2, 5-7, 9-12, 14, 17) | - |  |
|  |  | | Transportation, non-medical (including toll) | 7 | (5-7, 9-12) | - |  |
|  |  |  | Transportation, medical | 5 | (5, 6, 9, 10, 12) | - |  |
|  |  |  | Ambulance transportation | 1 | (2) | - |  |
|  |  |  | Not specified | 3 | (2, 14, 17) | - |  |
|  | (Other) out-of-pocket costs | | | 6 | (5, 7, 11, 12, 17, 18) | 3 | (1, 9, 14) |
|  |  | | Home adaptations and special equipment (toilet seat appliance, bathtub or shower appliance, stair lift) | 5 | (5, 7, 11, 12, 17) | - |  |
|  |  |  | Extra telephone costs | 3 | (5, 7, 12) | - |  |
|  |  |  | Childcare for babysitting, also for other children (during medical visits/hospitalization of diseased child) | 4 | (5, 7, 12, 17) | - |  |
|  |  |  | Caregivers’ accommodations (e.g. when child is hospitalized/receives injection) | 3 | (5, 7, 17) | 1 | (9) |
|  |  |  | Money spend on food during medical visits | 2 | (5, 7) | - |  |
|  |  |  | Parking (for hospital or other medical visits) | 3 | (5, 7, 17) | - |  |
|  |  |  | Special diets | 1 | (18) | - |  |
|  |  |  | Folk remedies (e.g. copper bracelets) | 1 | (18) | - |  |
|  |  |  | Not specified | - | - | 2 | (1, 14) |
|  | Social care services/home care/private and community services | | | 4 | (6, 10-12) | - |  |
|  |  | | Use of social care services (i.e. formal (paid) care) and professional caregivers, including home medical care | 2 | (6, 10) | - |  |
|  |  |  | Use of private and community services/domestic help | 2 | (11, 12) | - |  |
|  | School costs | | | 3 | (7, 12, 17) |  |  |
|  |  | | Special assistance at school or home tutors | 1 | (7) | - |  |
|  |  |  | Special school arrangements (e.g. special school, or extra set of books) | 1 | (12) | - |  |
|  |  |  | Educational support for the child or siblings (not further specified) | 1 | (17) | - |  |
| Productivity costs | Productivity loss of caregivers (including informal caregiving) | | | 11 | (2, 4-10, 12, 17, 20) | 3 | (1, 11, 14) |
|  |  | | Work/sick leave due to child’s illness, time lost due to health care appointments and/or due to informal caregiving (absenteeism) | 11 | (2, 4-10, 12, 17, 20) | 1 | (11) |
|  |  |  | Cease employment/early retirement | 3 | (6, 10, 17) | - |  |
|  |  |  | Reduced number of working hours | 1 | (17) | - |  |
|  |  |  | Presenteeism | 1 | (17) | - |  |
|  |  |  | Healthcare assistance due to the child’s JIA | 1 | (17) | - |  |
|  |  |  | Productivity losses, not specified | - |  | 2 | (1, 14) |
|  | Missed school days and productivity loss of patients | | | 5 | (6, 10-12, 17) | 3 | (1, 2, 7) |
|  |  | | Sick leave from school | 3 | (2, 12, 17) | 1 | (11) |
|  |  |  | Sick leave from work | 3 | (6, 10, 11) | - |  |
|  |  |  | Cease employment/early retirement | 3 | (6, 10, 11) | - |  |
|  |  |  | Impact on future employment ability | - |  | 3 | (1, 2, 7) |
|  | Missed school days of siblings | | | 1 | (17) | - |  |

**References:**

1. Luca NJ, Burnett HF, Ungar WJ, Moretti ME, Beukelman T, Feldman BM, et al. Cost-Effectiveness Analysis of First-Line Treatment With Biologic Agents in Polyarticular Juvenile Idiopathic Arthritis. Arthritis Care Res (Hoboken). 2016;68(12):1803-11.

2. Bernatsky S, Duffy C, Malleson P, Feldman DE, St Pierre Y, Clarke AE. Economic impact of juvenile idiopathic arthritis. Arthritis Rheum. 2007;57(1):44-8.

3. Shepherd J, Cooper K, Harris P, Picot J, Rose M. The clinical effectiveness and cost-effectiveness of abatacept, adalimumab, etanercept and tocilizumab for treating juvenile idiopathic arthritis: a systematic review and economic evaluation. Health Technol Assess. 2016;20(34):1-222.

4. Ungar WJ, Costa V, Hancock-Howard R, Feldman BM, Laxer RM. Cost-effectiveness of biologics in polyarticular-course juvenile idiopathic arthritis patients unresponsive to disease-modifying antirheumatic drugs. Arthritis Care Res (Hoboken). 2011;63(1):111-9.

5. Ens A, Lang B, Ramsey S, Stringer E, Huber AM. The financial burden of juvenile idiopathic arthritis: a Nova Scotia experience. Pediatr Rheumatol Online J. 2013;11(1):24.

6. Angelis A, Kanavos P, Lopez-Bastida J, Linertova R, Serrano-Aguilar P, Network B-RR. Socioeconomic costs and health-related quality of life in juvenile idiopathic arthritis: a cost-of-illness study in the United Kingdom. BMC Musculoskelet Disord. 2016;17:321.

7. Allaire SH, DeNardo BS, Szer IS, Meenan RF, Schaller JG. The economic impacts of juvenile rheumatoid arthritis. J Rheumatol. 1992;19(6):952-5.

8. Epps H, Ginnelly L, Utley M, Southwood T, Gallivan S, Sculpher M, et al. Is hydrotherapy cost-effective? A randomised controlled trial of combined hydrotherapy programmes compared with physiotherapy land techniques in children with juvenile idiopathic arthritis. Health Technol Assess. 2005;9(39):iii-iv, ix-x, 1-59.

9. Haapasaari J, Kautiainen HJ, Isomaki HA, Hakala M. Etanercept does not essentially increase the total costs of the treatment of refractory juvenile idiopathic arthritis. J Rheumatol. 2004;31(11):2286-9.

10. Kuhlmann A, Schmidt T, Treskova M, Lopez-Bastida J, Linertova R, Oliva-Moreno J, et al. Social/economic costs and health-related quality of life in patients with juvenile idiopathic arthritis in Europe. Eur J Health Econ. 2016;17 Suppl 1:79-87.

11. Minden K, Niewerth M, Listing J, Biedermann T, Schontube M, Zink A. Burden and cost of illness in patients with juvenile idiopathic arthritis. Ann Rheum Dis. 2004;63(7):836-42.

12. Minden K, Niewerth M, Listing J, Mobius D, Thon A, Ganser G, et al. The economic burden of juvenile idiopathic arthritis-results from the German paediatric rheumatologic database. Clin Exp Rheumatol. 2009;27(5):863-9.

13. Pohjankoski H, Latva K, Kautiainen H, Saila H, Klaukka T, Virta L, et al. First-year purchases of disease-modifying drugs of incident patients with chronic juvenile arthritis in Finland. Clin Exp Rheumatol. 2011;29(5):878-81.

14. Prince FH, de Bekker-Grob EW, Twilt M, van Rossum MA, Hoppenreijs EP, ten Cate R, et al. An analysis of the costs and treatment success of etanercept in juvenile idiopathic arthritis: results from the Dutch Arthritis and Biologicals in Children register. Rheumatology (Oxford). 2011;50(6):1131-6.

15. Thornton J, Ashcroft D, O'Neill T, Elliott R, Adams J, Roberts C, et al. A systematic review of the effectiveness of strategies for reducing fracture risk in children with juvenile idiopathic arthritis with additional data on long-term risk of fracture and cost of disease management. Health Technol Assess. 2008;12(3):iii-ix, xi-xiv, 1-208.

16. Thornton J, Lunt M, Ashcroft DM, Baildam E, Foster H, Davidson J, et al. Costing juvenile idiopathic arthritis: examining patient-based costs during the first year after diagnosis. Rheumatology (Oxford). 2008;47(7):985-90.

17. Shenoi S, Horneff G, Cidon M, Ramanan AV, Kimura Y, Quartier P, et al. The burden of systemic juvenile idiopathic arthritis for patients and caregivers: an international survey and retrospective chart review. Clin Exp Rheumatol. 2018.

18. Toupin-April K, Feldman DE, Zunzunegui MV, Descarreaux M, Malleson P, Duffy CM. Longitudinal analysis of complementary and alternative health care use in children with juvenile idiopathic arthritis. Complement Ther Med. 2009;17(4):208-15.

19. Seburg EM, Horvath KJ, Garwick AW, McMorris BJ, Vehe RK, Scal P. Complementary and alternative medicine use among youth with juvenile arthritis: are youth using CAM, but not talking about it? J Adolesc Health. 2012;51(2):200-2.

20. Rasu RS, Cline SK, Shaw JW, Hayes O, Bawa WA, Cifaldi MA. Impact of JIA on parents' work absences. Rheumatology. 2015;54(7):1177-85.
